# Primary follicular dendritic cell sarcoma of the urinary bladder: the first case report and potential diagnostic pitfalls

**DOI:** 10.1186/s13000-017-0625-4

**Published:** 2017-04-24

**Authors:** Guang-Jie Duan, You-Li Wu, Hui Sun, Lang Lang, Zhi-Wen Chen, Xiao-Chu Yan

**Affiliations:** 1 Institute of Pathology and southwest cancer center, Southwest Hospital, Third Military Medical University, No. 29, Gaotanyan street, Shapingba district, Chongqing, 400038 China; 2 Department of Urology, Southwest Hospital, Third Military Medical University, Chongqing, 400038 China

**Keywords:** Follicular dendritic cell sarcoma, Urinary bladder, Metastasis, Diagnostic pitfall, Treatment

## Abstract

**Background:**

Extranodal follicular dendritic cell sarcoma (FDCS) is a very rare malignancy with a variable clinical course. It is often not considered and has the potential to result in a misdiagnosis of other common sarcomas or sarcomatoid carcinomas. This is particularly true with the preoperative biopsy specimen, in which the tissue sample is often small.

**Case presentation:**

A case of FDCS in a 63-year-old woman, arising in the urinary bladder, a previously unreported site, is described. The patient presented with the typical clinical symptoms of a bladder cancer, and the morphology of the tumor was similar to a lymphoepithelioma-like carcinoma, ultimately resulting in it being misdiagnosed. The patient received radical cystectomy, without further radiotherapy or chemotherapy. Two years after operation, a metastatic tumor to the lung was found. The mass of the right main bronchus lumen was frozen and resected through bronchoscopy, and radiotherapy was performed. The patient has lived with the tumor since then.

**Conclusions:**

This paper presents the first FDCS occurring in the urinary bladder with metastasis to the lung and emphasizes potential diagnostic pitfalls.

## Background

Follicular dendritic cell sarcoma (FDCS) is a rare malignancy derived from follicular dendritic cells, which form a meshwork in lymphoid follicles and have the role of antigen capture and presentation [1, 2]. Although most lesions arise from lymph nodes, at least one-third occur in extranodal sites [2–4].

The conventional FDCS is composed of spindle to ovoid tumor cells with a varied architectural pattern in storiform (most common) or whorled (meningioma-like) bundles, fascicles, or diffuse sheets, sprinkled with some small mature lymphocytes [2, 3]. Although the cytologic features of these tumors are usually relatively bland, significant cytologic atypia may be found in some cases. The epithelioid cytomorphology is less frequently observed. Immunohistochemistry (IHC) is indispensable for confirming the diagnosis. The tumor cells are commonly positive for CD21, CD23, CD35, CXCL-13 and podoplanin (D2-40), the markers of normal follicular dendritic cells. Although the vast majority of these tumors are negative for cytokeratin, CD3, CD20, CD31, CD34, CD1a, CD79a, myeloperoxidase, and HMB-45, exceptionally, cytokeratin, CD45 or CD20 can be expressed [5–8]. Besides the conventional FDCS, the inflammatory pseudotumor-like variant of FDCS occurs almost exclusively in the liver or spleen. The neoplastic spindle cells, which are consistently associated with Epstein-Barr virus (EBV), are dispersed within a prominent lymphoplasmacytic infiltrate [9].

Despite having typical histopathologic features and unique immunophenotype, extranodal FDCS cases remain challenging to diagnose because it is very rare and often not considered, particularly when it occurs in an uncommon site or the histopathologic morphology is atypical [3]. An example of extranodal FDCS arising in the urinary bladder, a previously unreported site [4], is described. The patient presented with the typical clinical symptoms and imaging characteristics of bladder cancer, and the tumor had the features of a lymphoepithelioma-like carcinoma, which made the diagnosis difficult, especially in the preoperative biopsy specimen. This paper emphasizes potential diagnostic pitfalls and increases awareness of the existence of FDCS in this rare anatomic region.

## Case presentation

**Fig. 1:**
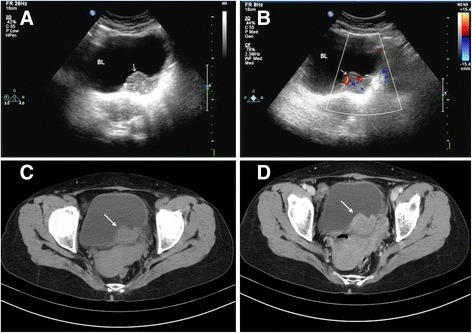
Abdominal ultrasound examination indicates a 43×28 mm slightly enhanced echo on the *left* side of the bladder wall (**a**). Color Doppler Flow Imaging detects a strip-like blood flow signal and records the pulsatile flow spectrum (**b**). Pelvic-enhanced computed tomography examination shows nodular soft tissue shadow at the left posterior wall of the bladder (**c**), which was obviously enriched after enhancement (**d**)

A 63-year-old woman presented to our hospital due to painless gross hematuria lasting for 20 days. She complained of gross hematuria and occasional blood clots, without symptoms of frequent micturition, urgency, odynuria and fever. Physical examination showed mild tenderness, no palpable mass and no other abnormalities. Urine examination indicated red blood 1 +, and the remaining biochemical indexes and the electrolyte indicators of liver and kidney function, routine blood and stool levels were all within the normal range. An abdominal ultrasound examination indicated a 43×28 mm slightly enhanced echo on the left side of the bladder wall. Color Doppler Flow Imaging (CDFI) detected strip-like blood flow signal and recorded pulsatile flow spectrum, RI = 0.58 (Fig. 1a, b). Pelvic enhanced computed tomography (CT) examination showed a nodular soft tissue shadow at the left posterior wall of bladder, which presented obvious enrichment after enhancement (Fig. 1c, d). There were no signs of pelvic effusion,and no swollen lymph nodes were observed in the abdominopelvic cavity. All clinical studies pointed to a bladder carcinoma of the left posterior wall.

**Fig. 2:**
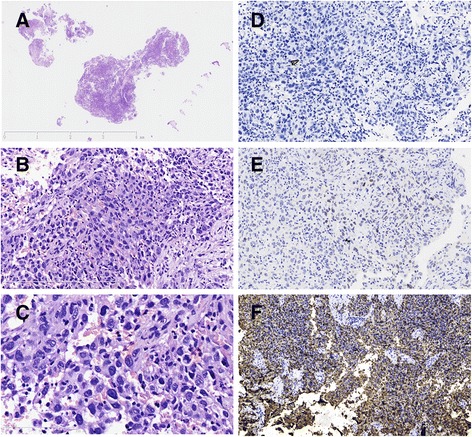
A low-power view of tumor tissue by transurethral cystoscopy and biopsy (**a**), HE staining reveals that round and polygonal epithelioid-like cells with a sheet or nest-like distribution (**b**), A high-power view shows the obvious cytologic atypia and atypical mitoses of the tumor cells (**c**, *arrows*). Immunohistochemical staining shows tumor cells are negative for CK (**d**), scattered positive for P63 (**e**), while diffuse positive for CD21 (**f**)

To establish a tissue diagnosis, transurethral cystoscopy and biopsy were performed, showing a 4×3 cm cauliflower-like new growth with a wide base at the left bladder wall, and there were no stones or foreign objects. Microscopically, hematoxylin and eosin staining revealed round and polygonal epithelioid cells with sheet or nest-like distributions, scattered neutrophils and lymphocyte infiltration. These epithelioid cells had obvious cytologic atypia, different-sized hyperchromatic nuclei and indistinct nucleoli. Atypical mitoses were easily observed (Fig. 2a-c). Thus, infiltrating urothelial carcinoma was the initial consideration. However, immunohistochemical staining showed that the tumor cells were negative for CK(AE1/AE3), CK7 and CK20, and only scattered superficial cells were positive for P63 (Fig. 2d, e), thus the primary consideration was questioned. By combining imaging characteristics with tissue morphology, malignant melanoma was considered next. However, further immunohistochemical staining showed that the tumor cells were negative for HMB45, melan-A and S-100, diffusely positive for vimentin, and focally positive for EMA. On account of little remaining biopsy tissue, a malignant tumor of the bladder, not further classifiable, was diagnosed, and a possibly poorly differentiated urothelial carcinoma was considered, thus surgical resection was recommended.

After a preoperative discussion and obtaining the patient’s informed consent, radical cystectomy and orthotopic ileal neobladder were performed. The intraoperative exploration indicated no adhesions between the bladder and abdominal wall, and no palpable lymph nodes were found in the abdominopelvic cavity and bilateral iliac peri-vessels. The resected specimens included the bladder, uterus, bilateral adnexae and appendix. The size of the bladder was 7.5×5.5 cm, and a cauliflower-like new growth, ranging from 3.5 to 3 cm in size and accompanied by partial superficial erosion, was observed on the left bladder wall.

**Fig. 3:**
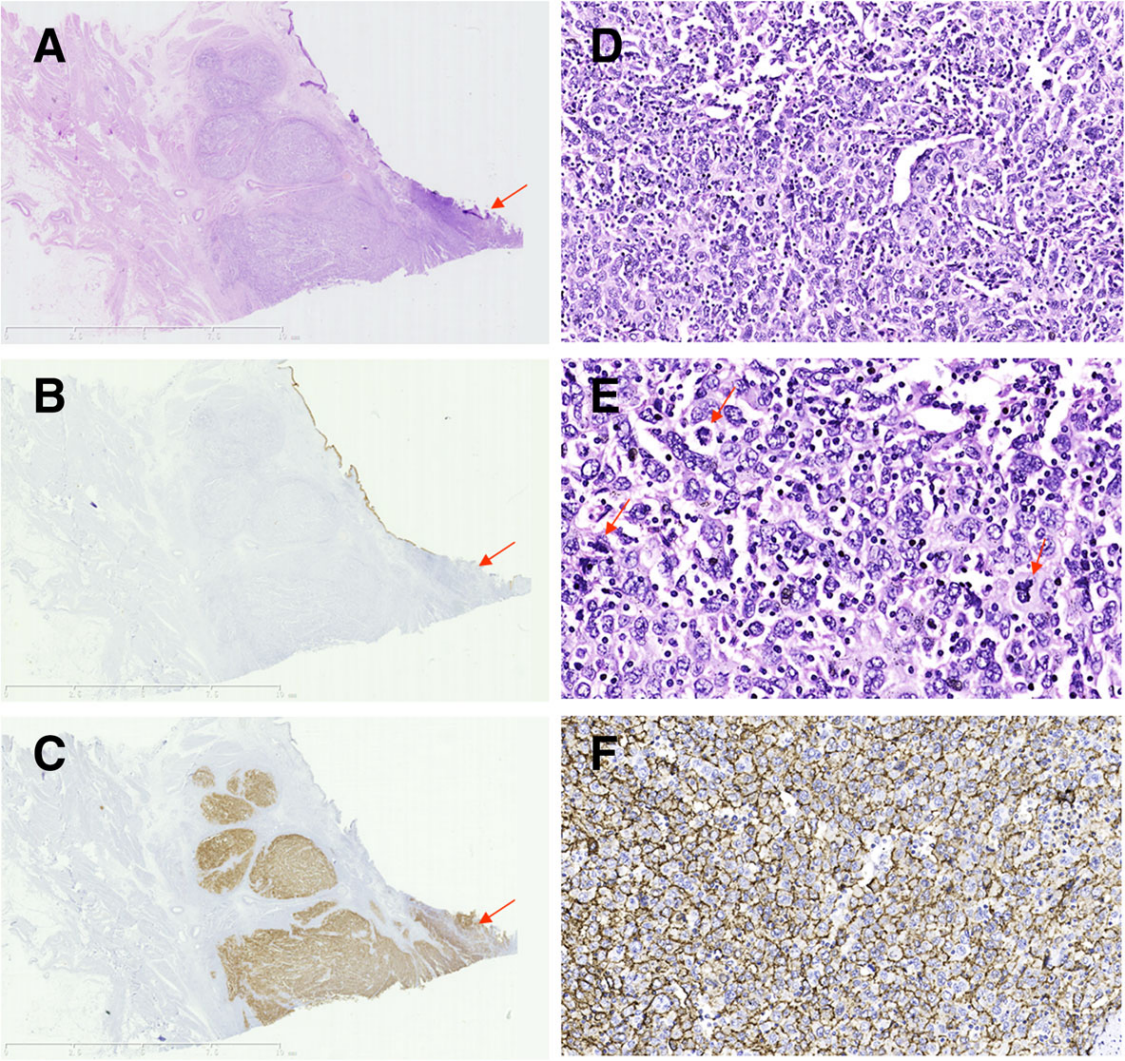
A low-power view of the tumor tissue by surgical resection reveals that the tumor cells are mainly located in the lamina propria and seem to be transitional with the surface mucosa (*arrows*) and infiltrates the muscular layer (**a**), The tumor cells are negative for CK staining (**b**) and diffusely positive for CD21 (**c**, **f**) by immunohistochemistry. The tumor cells are round, oval or polygonal with indistinct cellular borders and sprinkled with small lymphocyte infiltration, part of which had a syncytial-like appearance (**d**). Atypical mitoses are frequently observed (**e**, *arrows*)

Microscopically, the tumor cells were mainly located in the lamina propria, which seemed to be transitional with surface mucosa and infiltrated into the muscularis propria in a downward direction (Fig. 3a). The tumor cells were round, oval or polygonal with lightly eosinophilic cytoplasm and indistinct cellular borders, some of which had a syncytial-like appearance. The nuclei were round or ovoid with vesicular chromatin and prominent nucleoli. Atypical mitoses were frequently observed (Fig. 3d, e). The tumor cells were arranged in a diffuse sheet or nest-like pattern, among which some mature small lymphocytes and scattered multinucleated tumor giant cells were visible. Partial hemorrhage and necrosis were also observed. Cystitis glandularis and low grade atypia were found in the bladder mucosa adjacent to the tumor. The lymph nodes of the pelvic cavity showed reactive hyperplasia.

Combining the above morphological characteristics, a rare type of urothelial carcinoma (lymphoepithelioma-like carcinoma) was firstly considered. However, the immunohistochemical staining showed results similar to the preoperative biopsy: the tumor cells were uniformly negative for CK(AE1/AE3) (Fig. 3b), CK7, CK20, P63 (while the remaining superficial urothelial cells were positive), focally positive for EMA, and positive for vimentin and P53. The Ki-67 labeling index was approximately 50%. Thus, urothelial carcinoma was excluded.

By further carefully observing the pathologic morphology, as there was the infiltration of mature small lymphocytes among the tumor tissue, a scattered distribution of multinucleated tumor giant cells, as well as focal cystitis glandularis around the tumor lesions, we considered the case to be a very rare FDCS, which had not yet been reported in the bladder. Other possible differential diagnoses also included anaplastic large cell lymphoma, metastatic dysgerminoma and diffuse large B-cell lymphoma. Further immunohistochemical staining showed that the tumor cells were positive for CD21 (Fig. 3c, f), CD23, CD35, CXCL-13, focally positive for D2-40, and negative for CD3, CD20, CD30, CD15, ALK1, PLAP, CD117, CD38 and EGFR. In addition, the in situ hybridization for EBV-encoded RNA was also negative. Primary FDCS of the bladder was eventually diagnosed. The immunohistochemical staining for the remaining preoperative biopsy tissues also showed that the tumor cells were reactive to CD21, CD23, and CD35(Fig. 2f), supporting the diagnosis of FDCS of bladder. Because of its rarity, this case had also further been reviewed and confirmed by the hematopathologists and GU experts in China.

Postoperative radiotherapy and chemotherapy were recommended, but the patient refused further treatment. Five months after the operation, an abdominal ultrasonography and CT examination revealed no signs of tumor recurrence and metastasis, and the patient was lost to follow-up.

**Fig. 4:**
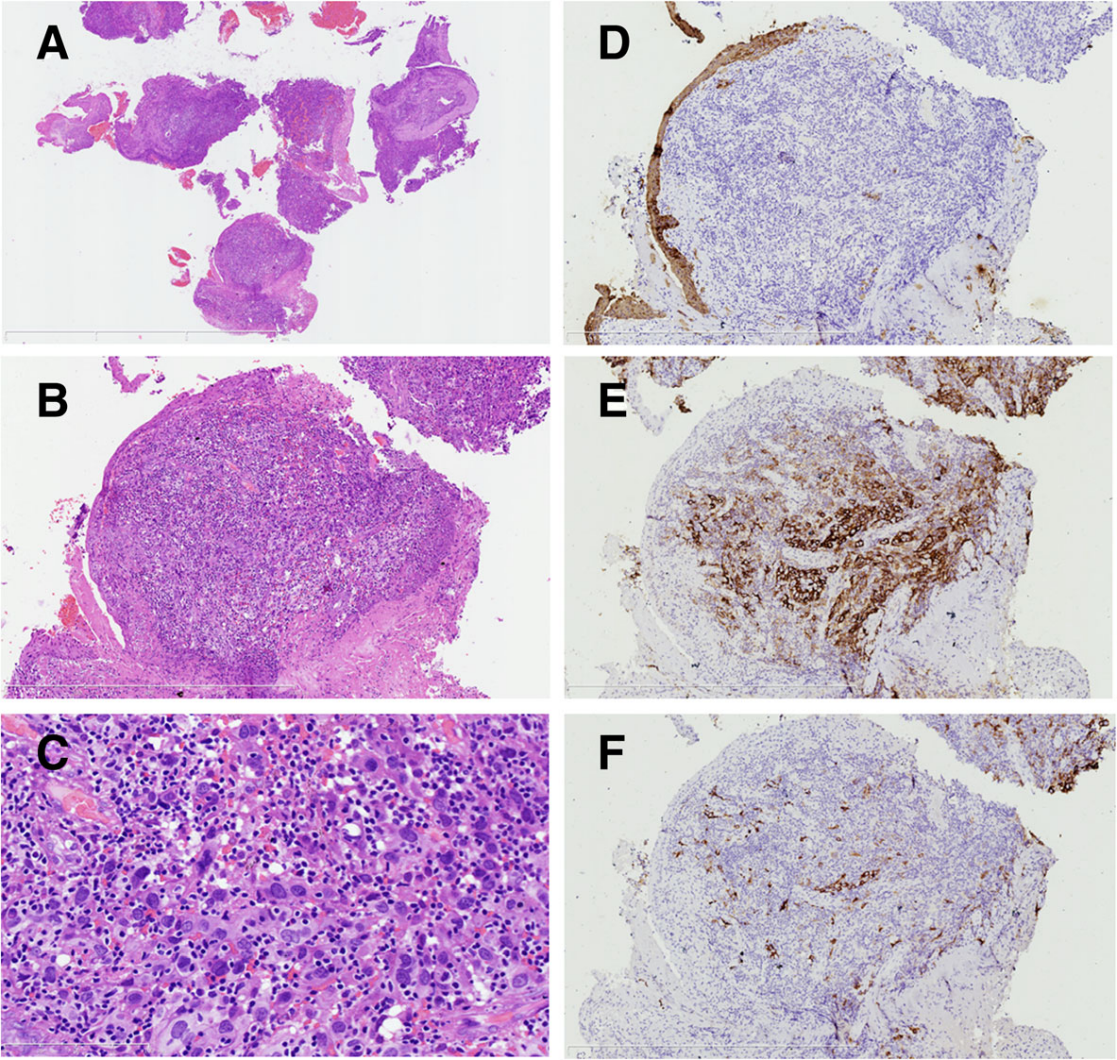
A low-power view of the tumor tissue by bronchoscopy and biopsy (**a**), metastatic tumor cells are beneath the respiratory epithelium of the trachea (**b**), A high-power view shows round and polygonal tumor cells with sprinkled small lymphocytes infiltration (**c**), similar to those occurring in the bladder. Immunohistochemically, the tumor cells are negative for CK (**d**), positive for CD21 (**e**) and focal positive for CD23 (**f**)

Two years after the operation, the patient came back to our hospital and complained of an intensely paroxysmal cough after cold, and occasionally having blood in the sputum. By bronchoscopy, a cauliflower-like mass protruding into the tracheal cavity was found in the middle of the trachea, and the right main bronchus opening was almost completely blocked. A biopsy of the mass was performed. Microscopically, the tumor cells similar to those occurring in the bladder were found beneath the respiratory epithelium of the trachea (Fig. 4a-c) that were negative for CK(AE1/AE3) (Fig. 4d), TTF-1 and P63, while were positive for CD21, CD23 (focal), and CD35 (focal) (Fig. 4e, f) by immunohistochemistry. The Ki-67 labeling index was approximately 50%. Thus, metastatic FDCS to the trachea was diagnosed.

To eliminate the risk of choking and to relieve dyspnea, considering the older age of the patient and her poor nutrition, the mass of the right main bronchus lumen was frozen and resected using bronchoscopy, and radiotherapy was performed. The patient has continued to live with the tumor.

## Discussion

FDCS, first documented in 1986 by Monda et al [1], is an unusual malignant neoplasm consisting of spindled to ovoid cells that have the morphologic and immunophenotypic features of follicular dendritic cells. Although approximately 60% of FDCS cases arise in lymph nodes, they can also occur in extranodal sites, such as the liver, lung, tonsil, spleen, soft tissue, digestive tract, retroperitoneum, nasopharynx, oral cavity, or breast [3, 4, 7–13]. However, the urinary bladder is a very unusual site for FDCS and, to the best of our knowledge, no case of primary bladder FDCS has previously been reported.

We present here a case of FDCS arising from the bladder, with epithelioid cytomorphology and obvious cytologic atypia. Thus, the case had been misdiagnosed as poorly differentiated urothelial carcinoma based on the preoperative biopsy. In our review of the literature, the reasons for a difficult diagnosis of this rare malignant condition are as follows:

First, the clinical symptoms and imaging characteristics of FDCS of bladder are very similar to those of bladder cancer. The initial symptoms of this patient included only painless gross hematuria for approximately 20 days, which is the typical symptom of bladder cancer. Moreover, preoperative imaging examinations indicated also a bladder carcinoma of the left posterior wall. Therefore, when we found the nested and lamellar distribution of atypical epithelial-like cells in the preoperative biopsy, we firstly considered it to be an invasive urothelial carcinoma. Even then, the IHC examination displayed epithelial-associated markers that were negative, and we still thought that the condition was a poorly differentiated urothelial carcinoma, as the latter may be accompanied by keratin expression loss.

Second, the histopathologic morphology of the surgically resected tumor was similar to that of lymphoepithelioma-like carcinoma, a rare subtype of urothelial carcinoma, so we did not initially consider the diagnosis of FDCS for the following reasons: (1) although a large number of small lymphocytes were infiltrating the tumor tissue, the tumor cells had an epithelial-like phenotype with remarkable atypia (which is a rare occurrence in FDCS); (2) tumor cells seem to be transitional with bladder mucosa, which is accompanied by low grade atypia, suggesting a possible urinary tract tumor origination; and (3) most importantly, FDCS is very rare, and had not been reported to occur in the bladder. Thus, we did not initially consider FDCS.

Extranodal FDCS is often not considered mainly also because the other sarcomas and sarcomatoid carcinomas are by far much more common, particularly when it occurs in visceral organs. So, the various other common spindle cell lesions should be excluded before the diagnosis of FDCS in the urinary bladder is made. The most important differential diagnosis of FDCS is sarcomatoid urothelial carcinoma, which can be distinguished by its tendency to have an infiltrative margin and more obvious nuclear pleomorphism. Expression of several cytokeratin and especially CK 34betaE12, CK 5/6 and p63 favors sarcomatoid carcinoma. In addition, immunoreactivity to FDC markers has not yet been reported in sarcomatoid urothelial carcinoma [14, 15]. Lymphoepithelioma-like urothelial carcinoma is excluded in a similar way.

Besides sarcomatoid carcinoma, inflammatory myofibroblastic tumor (IMT) or inflammatory pseudotumor (IPT) should also be taken into account when the neoplastic spindle cells are dispersed within a prominent lymphoplasmacytic infiltrate. IMT/IPT is usually diagnosed in the absence of prior surgery and no instance of metastasis has been documented. Coexpression of cytokeratin, muscle-specific antigens and ALK may be helpful for diagnosis [15–17]. In contrast, the diagnosis of postoperative spindle cell nodule (PSCN) of the bladder has been applied to those lesions histologically similar to IMT/IPT, which differs by involving older patients (usually those undergoing resections of urothelial carcinoma), smaller average size, presence of eosinophils and higher mitotic rate in some cases [16]. Finally, FDCS has to be separated from a variety of sarcomas, such as fibrosarcoma, malignant fibrous histiocytoma, leiomyosarcoma, rhabdomyosarcoma, malignant peripheral nerve sheath tumors and rarely interdigitating dendritic cell sarcoma, which also show a different immunophenotype [18].

Early reports suggested that FDCS should be an indolent tumor. We previously analyzed 39 cases of extranodal FDCS of the pharyngeal region and found that the overall recurrence, metastasis, and mortality rates were 23, 21, and 3%, respectively, also suggesting that this tumor should be a low-grade sarcoma [3]. However, further studies with longer follow-up periods, and the recognition of increased cytologic atypia, have indicated that FDCS is a more high-grade lesion, or at least an intermediate-grade malignancy often characterized by local recurrences and occasional distant metastases [2]. In this case, the tumor had obvious cellular atypia, necrosis and a high proliferative index with metastasis to the lung two years after the operation, which could be regarded as an intermediate-grade sarcoma.

FDCS has a variable clinical course, and the optimal treatment has not yet been defined. In addition to radical surgery, the efficacy of radiotherapy and chemotherapy remains unclear [18–20]. Chan et al suggested that adjuvant chemotherapy should be used for intra-abdominal FDCS owing to its high aggressiveness [12]. Through an analytic overview of 129 cases of FDCS and 55 cases of interdigitating dendritic cell sarcoma, De Pas et al noted that radical surgery alone was curative in approximately two thirds of these cases, and the data did not support the use of adjuvant treatments after radical excision [21]. Similarly, a pooled analysis of 462 reported cases of dendritic cell sarcoma (including 216 cases of FDCS with sufficient follow up data) also revealed that adjuvant radiotherapy had no statistically significant influence on overall survival of patients with early disease [4]. However, the latest pooled analysis of FDCS of the head and neck (97 cases) suggested that radiation and neck dissection may be beneficial for locoregional oncologic control [22]. For our patient, because the lesion was located in the abdomen and the tumor cells had an epithelial-like phenotype with remarkable atypia and necrosis, adjuvant chemotherapy was recommended. Unfortunately, the patient refused both radiotherapy and chemotherapy until the tumor metastasized to the lung.

## Conclusion

We report the first case of primary FDCS of bladder, which has typical clinical symptoms of a bladder cancer, and its morphology is similar to a lymphoepithelioma-like carcinoma. The above characteristics may make its diagnosis difficult, especially when the biopsy specimens are limited. Pathologists and urologists need be aware of this rare tumor, which should be included in the differential diagnosis of bladder tumors.

## Abbreviations

ALK: Anaplastic lymphoma kinase
CDFI: Color doppler flow imaging
CT: Computed tomography
DCS: Dendritic cell sarcoma
EBV: Epstein-Barr virus
EGFR: Epidermal growth factor receptor
EMA: Epithelial membrane antigen
FDCS: Follicular dendritic cell sarcoma
IHC: Immunohistochemistry
IMT: Inflammatory myofibroblastic tumor
IPT: Inflammatory pseudotumor
PLAP: Placental alkaline phosphatase
PSCN: Postoperative spindle cell nodule
RNA: Ribonucleic acid
